# Efficacy of Indigenous Bacteria in the Biodegradation of Hydrocarbons Isolated from Agricultural Soils in Huamachuco, Peru

**DOI:** 10.3390/microorganisms12091896

**Published:** 2024-09-14

**Authors:** Claudio Quiñones-Cerna, Alina Castañeda-Aspajo, Marycielo Tirado-Gutierrez, David Salirrosas-Fernández, Juan Carlos Rodríguez-Soto, José Alfredo Cruz-Monzón, Fernando Hurtado-Butrón, Wilmer Ugarte-López, Mayra Gutiérrez-Araujo, Medardo Alberto Quezada-Alvarez, Julieta Alessandra Gálvez-Rivera, Mario Esparza-Mantilla

**Affiliations:** 1 Laboratorio de Biotecnología e Ingeniería Genética, Facultad de Ciencias Biológicas, Universidad Nacional de Trujillo, Juan Pablo II Av., Trujillo 13008, Peru; 2 Departamento de Ingeniería Ambiental, Facultad de Ingeniería Química, Universidad Nacional de Trujillo, Juan Pablo II Av., Trujillo 13008, Peru; acastanedaa@unitru.edu.pe (A.C.-A.); mtiradog@unitru.edu.pe (M.T.-G.); wugartel@unitru.edu.pe (W.U.-L.); 3 Laboratorio de Citometría, Facultad de Ciencias Biológicas, Universidad Nacional de Trujillo, Juan Pablo II Av., Trujillo 13008, Peru; rsalirrosas@unitru.edu.pe (D.S.-F.); jrodriguezs@unitru.edu.pe (J.C.R.-S.); mgutierreza@unitru.edu.pe (M.G.-A.); 4 Departamento de Química, Facultad de Ingeniería Química, Universidad Nacional de Trujillo, Juan Pablo II Av., Trujillo 13008, Peru; jcruzm@unitru.edu.pe; 5 Laboratorio Multidisciplinario de Nanociencia y Nanotecnología “Oswaldo Sánchez Rosales”, Facultad de Ciencias Físicas y Matemáticas, Universidad Nacional de Trujillo, Juan Pablo II Av., Trujillo 13008, Peru; fhurtado@unitru.edu.pe; 6 Laboratorio de Investigación y Desarrollo en Ciencias Ambientales, Facultad de Ingeniería Química, Universidad Nacional de Trujillo, Juan Pablo II Av., Trujillo 13008, Peru; mquezada@unitru.edu.pe; 7 Escuela Profesional de Ciencias Biológicas, Facultad de Ciencias, Universidad Nacional de Piura, Juan Pablo II Av., Trujillo 13008, Peru; julietagalvezrivera@gmail.com; 8 Fundación Internacional para la Cultura y Ciencias de la Vida, Iquique 1100000, Chile; mrodrigount@yahoo.com

**Keywords:** biotechnology, bioremediation, mitigation, biodegradation, diesel, hydrocarbons

## Abstract

Pollution from crude oil and its derivatives poses a serious threat to human health and ecosystems, with accidental spills causing substantial damage. Biodegradation, using microorganisms to break down these contaminants, presents a promising and cost-effective solution. Exploring and utilizing new bacterial strains from underexplored habitats could improve remediation efforts at contaminated sites. This study aimed to evaluate the hydrocarbon biodegradation capacity of bacteria isolated from agricultural soils in Huamachuco, Peru. Soil samples from Oca crops were collected and bacteria were isolated. Biodegradation assays were conducted using diesel as the sole carbon source in the Bushnell Haas Mineral medium. Molecular characterization of the 16S rRNA gene identified four strains. Diesel biodegradation assays at 1% concentration were performed under agitation conditions at 150 rpm and 30 °C, and monitored on day 10 by measuring cellular biomass (OD_600_), with hydrocarbons analyzed by gas chromatography. The results showed *Pseudomonas protegens* (PROM2) achieved the highest efficiency in removing total hydrocarbons (91.5 ± 0.7%). Additionally, *Pseudomonas citri* PROM3 and *Acinetobacter guillouiae* ClyRoM5 also demonstrated high capacity in removing several individual hydrocarbons. Indigenous bacteria from uncontaminated agricultural soils present a high potential for hydrocarbon bioremediation, offering an ecological and effective solution for soil decontamination.

## 1. Introduction

Gasoil, or diesel, is a complex mixture of hydrocarbons obtained from the distillation of crude oil between 200 and 425 °C, primarily composed of 61% alkanes and 7.1% polycyclic aromatic hydrocarbons [1]. Diesel pollution and its recalcitrant components, such as saturated hydrocarbons and aromatics, represent a major anthropogenic pollution problem harmful to both human health and ecosystems, and cause considerable concern among governments and environmentalists [2]. This concern arises from the proven toxicity of these substances and the frequent spills resulting from accidents, leaks, pipeline interruptions or emissions of particulate matter resulting from combustion, which cause substantial environmental damage [3]. Consequently, the removal of these contaminants from the environment remains a priority. Although various strategies exist, such as chemical oxidation, thermal desorption, vapor extraction, and the use of electrolyzed catalytic and nanobubble systems, these methods often result in incomplete cleanup or the generation of undesirable by-products, in addition to being prohibitively expensive. [4].

Biodegradation offers a more promising approach by leveraging the metabolic capabilities of microorganisms to degrade contaminants, using them as sources of carbon and energy, offering a less costly and more ecological solution compared to chemical and physical methods [5]. This biological strategy can also be employed in bioremediation, with applications such as composting, bioventing, bioaugmentation, and the use of biopiles, among others [6]. Currently, a variety of hydrocarbon-degrading bacteria (HDB) have been reported (Table 1).

Other studies have investigated bacteria such as *Bacillus pumilus* and *Bacillus cereus*, which showed an efficiency of 84.15% in the degradation of total hydrocarbons after five weeks of incubation [11]. Another study identified and characterized bacterial strains and actinomycetes, such as *Pseudomonas proteolytica* and *Streptomyces sampsonii*, which demonstrated potential for the degradation of saturated hydrocarbons and polycyclic aromatic hydrocarbons [12]. These findings underscore the diversity and efficacy of indigenous microorganisms in the biodegradation of contaminants. Therefore, due to the notable potential of microorganisms for degradation, there is a growing interest in discovering and utilizing new bacterial strains from underexplored habitats where little microbial study has been conducted [13].

Typically, diesel-contaminated sites have been explored for hydrocarbon-degrading bacteria (HDB) to assess their ability to utilize hydrocarbons prior to biodegradation studies [14]; however, many microorganisms in natural, uncontaminated environments may possess hydrocarbon degradation pathways, with the terminal oxidation route being the most common, found in bacteria such as *Pseudomonas putida* KT2440, *Alcanivorax borkumensis* SK2 (T), and *Geobacillus thermodenitrificans* NG80-2 [15]. 

Recent studies have found a higher percentage of unclassified bacteria in contaminated soils compared to uncontaminated soils. For instance, Gao et al. [16] reported that diesel-contaminated agricultural lands showed high microbial abundance distributions when nitrogen amendments were applied.

In Peru, soil contamination by diesel hydrocarbons linked to the oil industry in recent years has caused numerous environmental incidents, mainly affecting cultivated areas and resulting in damage to flora and fauna [17]. *Pseudomonas* sp. has been isolated from soil contaminated by oil spills in the La Libertad region (Huamachuco), Peru, demonstrating a phenol-degrading capacity [18]; therefore, the elimination of pollution from oil and its derivatives through biological methods is crucial to protect human health and ecosystems, reducing costs, and improving the efficiency of environmental cleanup processes. The research and development of new bacterial strains capable of degrading diesel hydrocarbons can offer sustainable and effective solutions to this global problem [19].

This research investigated the hydrocarbon degradation performance in diesel samples in vitro using microbial cultures isolated from agricultural soils of Oca (*Oxalis tuberosa*) crops in Huamachuco, Peru. The microbial isolates were characterized by molecular analysis of the 16S rRNA gene, and hydrocarbon degradation was determined through profiles obtained by gas chromatography.

## 2. Materials and Methods

### 2.1. Sampling

**Figure 1:**
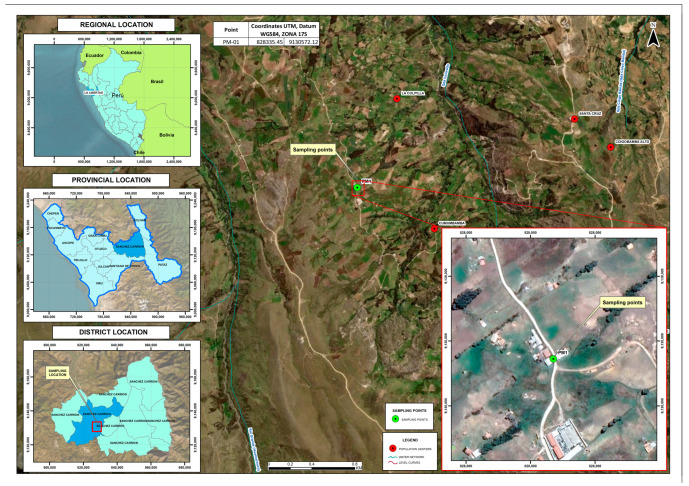
Agricultural soil sampling point in the Huamachuco region (Peru).

Agricultural soil samples were collected from an Oca (*Oxalis tuberosa*) crop in Huamachuco, Peru (Latitude -7.85494, longitude -78.02287) (Figure 1).

A soil sample (1000 g) was obtained at a depth of 30 cm, placed into a sterile container, and transported to the laboratory. Soil samples were sieved through a 2 mm sieve to remove stones and large debris. The samples were stored at 4 °C until processing.

### 2.2. Microbial Isolation

A total of 100 mL of Bushnell Haas Mineral (BHM) medium was prepared with the following composition (g/L): MgSO_4_.7H_2_O 0.2, CaCl_2_ 0.02, KH_2_PO_4_ 1, K_2_HPO_4_ 1, NH_4_NO_3_ 1, FeCl_3_ 100 µL/L at pH 6.3. The medium was sterilized at 121 °C for 15 min in a 250 mL flask [20]. Subsequently, 1% diesel (B5 S-50, Petroperú, Peru), previously filtered through a 0.22 µm membrane, was added. In the present study, B5 S-50 diesel was used since it contains 95% diesel and 5% bio-diesel, and is characterized by its low sulfur content [21]. One gram of the collected soil was enriched in 100 mL of BHM and incubated at 30 °C and 150 rpm for 7 days in an orbital shaker (Biobase, BJPX-200N, Jinan, China). Serial dilutions up to the tenth were then performed, and the last two dilutions (10^−8^ and 10^−9^) were surface-plated on Petri dishes containing Nutrient Agar (NA). Incubation was carried out at 30 °C for 48 h until the growth of colony-forming units (CFU). Microbial colonies with distinct morphologies were selected and subcultured in glass penicillin vials with slanted NA. The subcultures were incubated at 30 °C for 48 h and stored at 4 °C under refrigeration until use.

### 2.3. Selection of Hydrocarbon Degrading Bacteria

A total of 50 mL of sterile BHM medium was prepared, supplemented with 1% diesel as the sole carbon source. A total of 5% of a bacterial suspension from the isolated cultures was inoculated, adjusted to an optical density (OD) of approximately 1 at 600 nm, previously subcultured for 24 h of incubation and washed with a 0.85% NaCl solution by centrifugation (6000 rpm for 5 min) [22]. The treatments were incubated at 30 °C and 150 rpm for 10 days. Cellular biomass was monitored on days 5 and 10, and the percentage of hydrocarbon removal was determined.

### 2.4. Analytical Method 

Following the 8270e method for the measurement of semivolatile organic compounds, hydrocarbons were analyzed using a gas chromatograph (TRACE 1300, Thermo Scientific, Waltham, MA, USA) [23]. The method consisted of liquid–liquid extraction (LLE) for aqueous samples, where 8 mL of dichloromethane (GC grade, J.T. Baker, New York, NY, USA) was added to the sample, and the solvent was shaken vigorously for 2 min in a separatory funnel. The mixture was allowed to stand until the phases were completely separated. A total of 1 µL of the extracted sample was injected in split mode with an injector temperature of 250 °C, utilizing the T6-5MS column (Thermo Scientific, Waltham, MA, USA) with a length of 13 mm and a diameter of 0.25 mm. The total analysis time was 25 min. The transfer line and ionization source temperatures were maintained at 280 °C. A helium gas flow of 1 mL/min was employed. The percentage of hydrocarbon removal (HR%) was calculated using Equation (1) [24].

(1) $$\mathrm{HR}\%=\frac{\begin{array}{c} \mathrm{Sum}\;\mathrm{of}\;\mathrm{Initial}\;\mathrm{Area}\;\mathrm{under}\;\mathrm{each}\;\mathrm{individual}\;\mathrm{peak}-\mathrm{Sum}\;\mathrm{of}\;\mathrm{Final}\;\mathrm{Area}\;\mathrm{under}\;\mathrm{each}\;\\ \mathrm{individual}\;\mathrm{peak} \end{array}}{\mathrm{Sum}\;\mathrm{of}\;\mathrm{Initial}\;\mathrm{Area}\;\mathrm{under}\;\mathrm{each}\;\mathrm{individual}\;\mathrm{peak}}\;\times \;100\%\;$$

The optical density was determined using a UV-VIS spectrophotometer (SI Analytics—UviLine 9400, Mainz, Germany) at a wavelength of 600 nm. The specific growth rate (µ) was determined using linear regression of the decimal logarithm of optical density as a function of time during the exponential phase of microbial growth, through Equation (2) [25].

(2) $$µ\;\left(h^{-1}\right)=\frac{\Delta \log\left({\mathrm{OD}}_{600}\right)}{\Delta \;t\;\left(h\right)}$$

### 2.5. Identification of Selected Bacteria

The morphological characteristics of the colonies were determined using a stereoscope from a 24 h pure culture grown on nutrient agar. Additionally, their morphology was characterized through microscopy using Gram staining. 

The 24 h microbial cultures underwent a cell lysis process using the Quick-DNA™ Fungal/Bacterial Miniprep kit, following the manufacturer’s instructions. This process included the addition of a lysis buffer, incubation, and centrifugation to separate the DNA from other cellular components. PCR was performed to amplify the 16S ribosomal DNA segment. The PCR reaction was carried out in a total volume of 50 μL, which included 25 μL of PCR master mix, 1 μL of each primer 27F (5′-AGAGTTTGATCCTGGCTCAG-3′) and 1492R (5′-TACGGYTACCTTGTTACGACTT-3′), 2 μL of template DNA, and 21 μL of nuclease-free water, according to the protocol described by Tejada et al. [26]. The PCR product was loaded onto a 1.5% agarose gel prepared with TBE buffer and stained with ethidium bromide. Electrophoresis was conducted at 100 V for 45 min. The gel was visualized under UV light to confirm the presence and expected size of the amplified product. The PCR product was sequenced by capillary electrophoresis by Macrogen (Chile). The partial 16S rDNA sequence obtained was analyzed using MEGA X software. Sequence alignments were performed and a phylogenetic tree was constructed to determine the evolutionary relationships between the sequences. The obtained sequences were compared with those available in the EzBioCloud (accessed on 1 June 2024: https://www.ezbiocloud.net) database to identify and classify the microbial species [27].

### 2.6. Statistical Analysis

All experiments were carried out in triplicate, the data obtained from the hydrocarbon concentration were analyzed by analysis of variance (ANOVA), and the post hoc test was applied using the Tukey test, using the Origin 2018 v95E software package.

## 3. Results 

### 3.1. Chromatographic Characterization of Diesel

**Figure 2:**
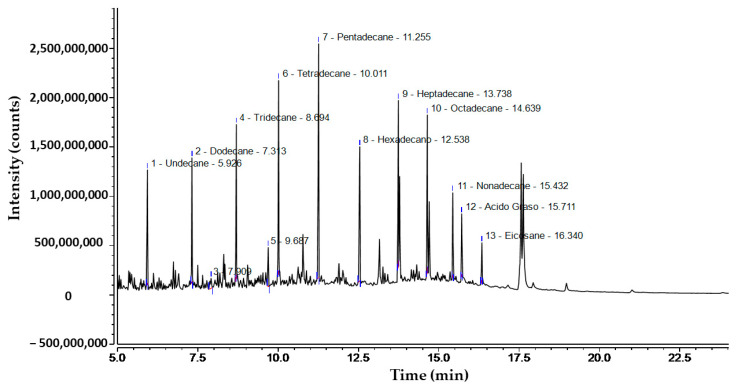
Chromatographic analysis of commercial diesel oil B5 S-50 used for the study.

The chromatogram of commercial diesel oil, shown in Figure 2, reveals the presence of various hydrocarbons, with distinct peaks corresponding to undecane (C11), dodecane (C12), tridecane (C13), tetradecane (C14), pentadecane (C15), hexadecane (C16), heptadecane (C17), octadecane (C18), nonadecane (C19), and eicosane (C20). The retention times for these compounds range from 5.92 to 16.34 min. Additionally, the presence of fatty acids at a retention time of 15.71 min suggests the influence of biodiesel compounds in the sample. The height and shape of the peaks on the chromatogram indicate the relative concentration of each compound present in the sample. The most abundant compounds, as inferred from the peak intensities, are pentadecane and tetradecane. These results reflect a homologous series of alkanes from C11 to C20, suggesting that the sample is predominantly composed of hydrocarbons. The accurate identification of these compounds was based on the retention times and fragmentation patterns in the mass spectrum, which is essential for understanding the chemical nature of the analyzed sample.

### 3.2. Characterization of the Isolated and Selected Bacteria

**Figure 3:**
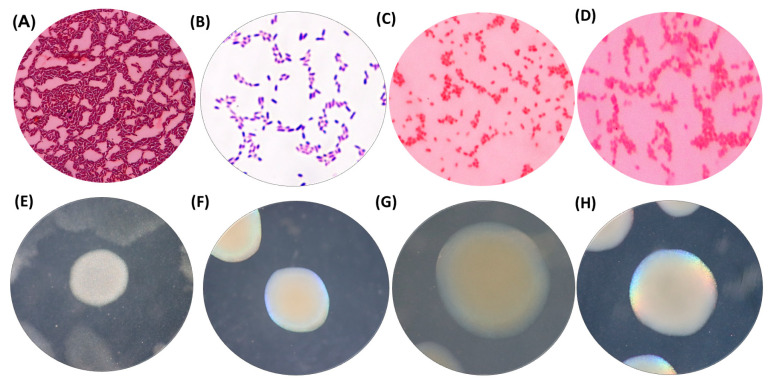
Morphological characteristics of the microbial cultures isolated from diesel-enriched agricultural soil: PROM1 (**A**,**E**), ClyRoM5 (**B**,**F**), PROM2 (**C**,**G**), and PROM3 (**D**,**H**).

Four types of pure microbial cultures were isolated from agricultural soil in Huamachuco, Peru, which had been enriched with diesel as the sole carbon source. These isolates were designated as PROM1, PROM2, PROM3, and ClyRoM5. Microscopic characterization revealed that ClyRoM5, PROM2, and PROM3 were Gram negative, whereas PROM1 was Gram positive. All isolates exhibited a bacillary shape (Figure 3). 

Regarding the macroscopic characteristics of their colonies, PROM2 displayed elevated, opaque, medium-sized colonies with circular and mucous edges; PROM3 exhibited elevated, smooth, medium-sized colonies with circular and mucous edges; PROM1 showed flat, medium-sized colonies with irregular and smooth edges; and ClyRoM5 presented pinpoint, medium-sized, circular and mucous colonies (Figure 3).

The molecular characterization based on 16S rRNA gene sequencing of the isolated microbial strains revealed that PROM1, PROM2, PROM3, and ClyRoM5 exhibited highly significant pairwise similarity ranging from 99.18 to 100% when aligned with the sequences of *Priestia flexa* NBRC 15715, *Pseudomonas protegens* CHA0, *Pseudomonas citri* OPS13-3, and *Acinetobacter guillouiae* OPS13-3, respectively. Furthermore, the amplicons obtained from PROM1, PROM2, PROM3, and ClyRoM5 had base pair (bp) sizes of 990, 725, 1343, and 1341, respectively, and the nucleotide sequences were deposited in the National Center for Biotechnology Information (NCBI) database with accession numbers PP886133, PP886146, PP886148, and PP892527 (Table 2). 

**Figure 4:**
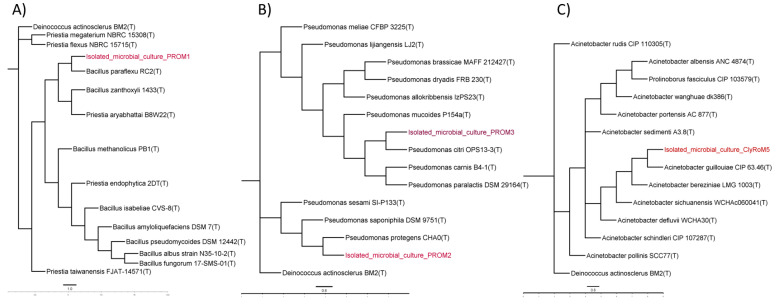
Phylogenetic tree inferred using the neighbor-joining method with a bootstrap of 1000 replicates of the isolated microbial culture PROM1 (**A**), PROM2/PROM3 (**B**), and ClyRoM5 (**C**). Evolutionary distances were calculated using the Tamura 3-parameter nucleotide substitution model. *Deinococcus actinosclerus* BM2(T) was selected as the outgroup.

Figure 4 illustrates the phylogenetic tree constructed from the nucleotide sequences of the isolated microbial cultures PROM1, PROM2, PROM3, and ClyRoM5, aligned with type strains (T) sequences obtained from the EzBioCloud database. It can be observed that PROM3 and PROM2 clustered together in the same clade with an evolutionary distance of less than 0.004 and 0.00 with *Pseudomonas citri* and *Pseudomonas protegens*, respectively, suggesting they share a more recent common ancestor compared to the other samples, which coincides with the observed lower evolutionary distance in the data. PROM1 was found on a more distant branch compared to the other three samples, indicating greater genetic divergence. This greater phylogenetic distance suggests that PROM1 has an older common ancestor with the other samples but presents a better branch distance of 0.004 with *Priestia flexa*. Meanwhile, ClyRoM5 is located on a separate branch, although still relatively close to PROM2 and PROM3, indicating moderate genetic similarity, consistent with the observed intermediate evolutionary distance.

### 3.3. Diesel Biodegradation Tests

**Figure 5:**
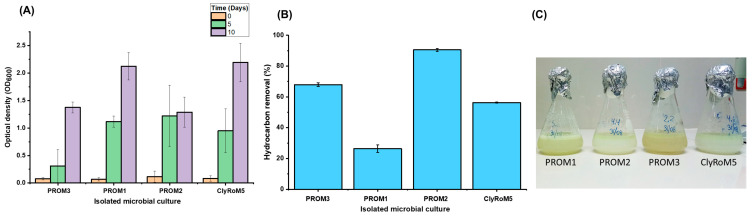
Accumulation of cell biomass (OD_600_) in the medium with diesel by isolated microbial cultures (**A**), percentage of hydrocarbons removal (**B**), and determination of the final turbidity after a 10-day incubation using diesel as the sole carbon source (**C**).

Figure 5 illustrates experimental assays of hydrocarbon biodegradation by the strains PROM1, PROM2, PROM3, and ClyRoM5. PROM2 shows the highest efficiency in hydrocarbon removal with 91.5 ± 0.7% over 10 days at 150 rpm at 30 °C, indicating a high biodegradation capacity. This result is notable given its intermediate biomass growth of 1.288 (OD_600_). PROM3 and ClyRoM5 exhibit moderate efficiencies of 67 ± 1.41% and 57.5 ± 0.71%, respectively. Although ClyRoM5 showed high biomass growth, its hydrocarbon removal capacity was lower compared to PROM2. 

Table 3 presents the statistical analysis that PROM1 has significantly lower values than PROM3, with a probability (p) less than 0.05. PROM2 significantly outperforms both PROM1 and PROM3, with mean differences of 65.38 and 23.87, respectively, and *p* < 0.05. ClyRoM5 has significantly higher values than PROM1, with a mean difference of 3.13 and a probability of *p* < 0.05, and significantly lower values than PROM2, with a mean difference of −34.1 and a probability of 0.0. However, it does not present significant differences with PROM3, with a probability of 0.42. The results indicate that PROM1 is significantly lower than PROM3, while PROM2 significantly outperforms both PROM1 and PROM3. ClyRoM5 shows significant differences with PROM1 and PROM2 but not with PROM3, suggesting a similar behavior between ClyRoM5 and PROM3.

PROM3 exhibited specific growth rate (µ), with a value of 0.73 ± 0.2 (h^−1^) with 68% total hydrocarbon removal, suggesting a strong link between cell proliferation and hydrocarbon degradation capacity. On the other hand, PROM1 presents a remarkably low µ, of only 0.06 ± 0.001 (h^−1^). This slow growth is differed by a low percentage of hydrocarbon removal, reaching only 27%. These results indicate that PROM1 has a limited capacity both to grow and to degrade hydrocarbons, making it the least efficient among the cultures analyzed. The PROM2 culture shows a growth-to-degradation ratio with a µ of 0.60 ± 0.02 (h^−1^), in turn achieving the highest percentage of total hydrocarbon removal, reaching 92%. This suggests that, although it does not have the highest µ, PROM2 is extremely effective in hydrocarbon degradation. Finally, ClyRoM5, with a µ of 0.67 ± 0.04 (h^−1^), removes 57% of the total hydrocarbons, despite its good growth rate. The removal percentage is moderate compared to PROM2.

**Figure 6:**
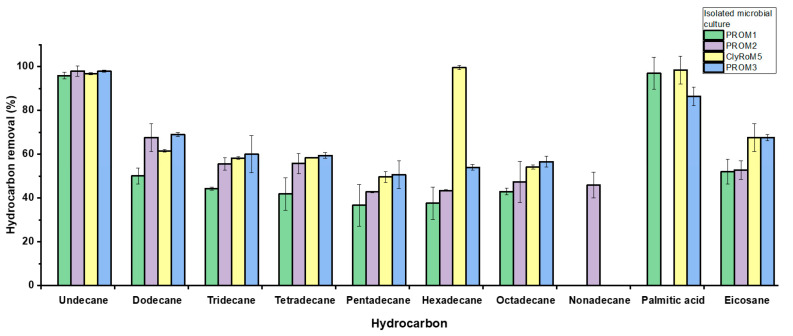
Percent removal of individual hydrocarbons from B5 S-50 diesel by the isolated microbial cultures over 10 days at 150 rpm and 30 °C.

Figure 6 shows the percentage of removal of various individual hydrocarbons from B5 S-50 diesel by isolated microbial cultures (PROM1, PROM2, ClyRoM5, and PROM3). All cultures showed high removal efficiency (95.89–97.95%) for Undecane (C11), indicating high consistency in the biodegradation of this compound. Dodecane (C12) removal varied significantly among the cultures, with *Pseudomonas protegens* PROM2 and *Pseudomonas citri* PROM3 showing higher efficiency (67.56 and 68.98%, respectively) compared to *Priestia flexa* PROM1 (50.04 ± 3.67%). *Pseudomonas citri* PROM3 demonstrated the highest efficiency in the removal of Tridecane (C13), Tetradecane (C14), and Pentadecane (C15), with removal rates of 60.01 ± 8.45%, 59.42 ± 1.35%, and 50.64 ± 6.41%, respectively. *Acinetobacter guillouiae* ClyRoM5 showed high removal (99.46 ± 0.95%) of Hexadecane (C16), significantly higher than the other cultures. *Pseudomonas citri* PROM3 showed the highest efficiency in the removal of Octadecane (C18) and Eicosane (C20), with values of 56.60 ± 2.40% and 67.64 ± 1.43%, respectively, followed closely by ClyRoM5. In contrast, *Pseudomonas protegens* PROM2 was the only culture that showed significant activity in the removal of Nonadecane (C19), with 45.87 ± 5.87%, while *Acinetobacter guillouiae* ClyRoM5 had the highest efficiency in the removal of Palmitic acid, with 98.32 ± 6.37%, surpassing *Priestia flexa* PROM1.

## 4. Discussion

According to the standards, most of the n-alkanes of interest for biodegradation testing in this study were identified (Figure 2). This is crucial to evaluate the degradation capacity of different bacterial strains and predict their efficiency in the bioremediation of soils contaminated with hydrocarbons [28]. The chromatogram reported in the study showed distribution patterns and levels similar to those analyzed by Yang et al. [29], who also found predominant hydrocarbons such as n-C15, n-C14, and n-C16 from a sample of biodiesel in hexane. The presence of these components could influence the biodegradation time of diesel, as the stability of alkanes and the length of their carbon chains may require specific conditions and extended end periods to achieve significant degradation [30].

Four microbial cultures capable of growing from diesel as the sole carbon source were isolated and selected (Table 2). Additionally, according to the literature, *Pseudomonas protegens* species have been similarly reported in riverbank soils as rhizospheric bacteria [31]; *Priestia flexa* VL1 has been reported in tannery effluent-contaminated soils as a potential bioremediator [32]; likewise, *Pseudomonas citri* has been reported in citrus rhizosphere soil [33]. *Acinetobacter guillouiae* has not been reported in the literature in the removal of hydrocarbons; however, in the case of the *Acinetobacter* group, species have been reported as potential agents in the degradation of crude diesel [34]. Another study also reported the presence of two bacteria from the *Acinetobacter* and *Pseudomonas* groups capable of degrading diesel, as Dohare et al. [35] isolated *Acinetobacter pittii* ED1 and *Pseudomonas aeruginosa* BN, which optimally degrade diesel at 30 °C, with a pH of 7.0 and 1% diesel. Similarly, Palanisamy et al. [36] highlights another species, *Acinetobacter baumannii*, in diesel-contaminated soils, finding that this microorganism can effectively degrade diesel under optimal cultivation conditions; therefore, this study reports for the first time the involvement of the species *Acinetobacter guillouiae* in diesel degradation.

The increases in optical density of the four isolated microbial cultures (Figure 3) indicated they utilized diesel as a growth source, energy, and biomass increase, reflected in the turbidity of the culture medium [37]. According to the categorization described by Talaiekhozani et al. [38], low growth is indicated by an OD_600_ range of 0.21–0.40, moderate growth by a range of 0.41–0.60, high growth by a range of 0.61–0.80, and excellent growth by a range of 0.81–1.00. Therefore, the results obtained showed that all four isolated microbial cultures exhibited excellent growth, with OD_600_ values exceeding 1, falling within a range of 1–2. The results obtained in the study of the hydrocarbon-degrading isolates PROM1, PROM2, PROM3, and ClyRoM5 can be compared with similar studies highlighting the capabilities of different bacteria in hydrocarbon degradation. For example, *Acinetobacter calcoaceticus* CA16 demonstrated a remarkable growth capacity in the presence of diesel, reaching an OD_600_ of approximately 1.12 after 20 days, emphasizing its efficiency in utilizing diesel as a carbon source [39]. Another study evaluated the efficiency of *Stenotrophomonas maltophilia* and found that this bacterium not only grew well in the presence of diesel but also produced biosurfactants that facilitate hydrocarbon degradation [40]. Additionally, strains of *Pseudomonas aeruginosa* have been shown to efficiently degrade petroleum hydrocarbons due to the production of rhamnolipids, which enhance the solubility and bioavailability of the contaminants [41]. 

The maximum percentage of hydrocarbon removal (91.5 ± 0.7%) in this study surpasses that of Chaudhary et al. [42], who obtained 79.0% and 85.4% degradation of diesel hydrocarbons (C18, C20 and C22) using *Acinetobacter* sp. K-6, and Panda et al. [43], who reported a degradation of 49.93% of diesel for 20 days at 150 rpm at 37 °C using *Pseudomonas* sp. Moreover, the isolated *Pseudomonas protegens* PROM2 achieved a degradation efficiency comparable to that of a microbial consortium reported by Otiniano et al. [44], with a value of 94.77%, using 10% inoculum for 5% diesel. Similarly, previous studies have identified *Pseudomonas protegens* as an effective bioremediation agent for toxic metal contamination [45]; however, little has been found regarding its action against hydrocarbon biodegradation as demonstrated in this study.

Figure 5 shows no direct correlation between the percentage of hydrocarbon removal and cell growth (OD_600_) in the strains studied. Although some strains achieved high efficiency in hydrocarbon removal, their cell growth did not increase proportionally, suggesting that hydrocarbon degradation is not directly related to cell proliferation and that they could be using other metabolic or energetic mechanisms not reflected in an increase in biomass. As Wang et al. [15] highlighted, that the efficiency of degradation may depend on specific interactions within the microbial communities and the presence of specialized enzymes (hydroxylases, aldehyde dehydrogenase, dioxygenase, alcohol dehydrogenase and aldehyde dehydrogenase). This lack of correlation could be due to factors such as the use of other hydrocarbons available in the medium, the accumulation of intermediate products, or the specific metabolic adaptation of each strain [46].

Table 3 shows that PROM2 is significantly more efficient in hydrocarbon removal compared to PROM1 and PROM3, as demonstrated by the mean differences of 65.38 and 23.87, respectively, both with a probability value (*p* < 0.05). This suggests that *Pseudomonas protegens* PROM2 has a superior degradation capacity. However, when comparing the degradation capacity of *Acinetobacter guillouiae* ClyRoM5 and *Pseudomonas citri* PROM3, the results indicate comparable efficiencies, suggesting that both strains could be viable candidates to form effective consortia. Previous studies have shown that microbial consortia can enhance hydrocarbon degradation due to the synergy between different species [47].

A µ of 0.05–0.31 h^−1^ was obtained from the four isolated microbial cultures. These results surpass those obtained by Sharma et al. [48], who reported a µ value of 0.052 h^−1^ using *P. aeruginosa* at 37 °C and 180 rpm over approximately 25 days in the removal of crude oil. Additionally, they differ from the results studied by Azzahra et al. [49], who reported specific growth rate (µ) values ranging from 0.0440 to 0.0952 h^−1^ using a consortium of *Acetobacter tropicalis* and *Lactobacillus casei* to degrade TPH in both SMSS liquid medium and artificial seawater. Furthermore, these results differ from those obtained by Zannotti et al. [50], who reported a specific growth rate of 0.0297 h^−1^ for *Marinomonas sp*. at 22 °C with 1% diesel.

Microbial cultures varied in their efficiency and accuracy for removing hydrocarbons from B5 S-50 diesel, with *Pseudomonas citri* PROM3 and *Acinetobacter guillouiae* ClyRoM5 standing out for their versatility and overall effectiveness, while *Pseudomonas protegens* PROM2 and *Priestia flexa* PROM1 excelled in the removal of specific compounds. These results are comparable to studies on diesel hydrocarbon biodegradation using mixed cultures of *Bacillus subtilis* InaCC B289 and *Pseudomonas aeruginosa* InaCC B290, achieving 57.56% degradation, showing effective removal of octadecane (62.78%) and Eicosane (42.23%) [51]. Another study found that the *Acinetobacter* sp. JYZ-03 strain efficiently degraded diesel n-alkanes, reaching up to 84.05% efficiency in long-chain n-alkanes, although it showed lower efficiency in undecane degradation (48.78%) [52].

Likewise, analyzing the impact of different environmental factors, such as pH, temperature, and nutrient availability, on the efficiency of hydrocarbon biodegradation by indigenous bacteria obtained in this study, could help optimize conditions for bioremediation in specific sites. In turn, exploring new bioremediation technologies, such as biostimulation with natural surfactants and bioaugmentation, could improve the degradation of recalcitrant contaminants in future research [53].

## 5. Conclusions

This study conducted in Huamachuco, Peru, revealed the significant potential of native bacterial strains for hydrocarbon biodegradation in agricultural soils. Four isolated strains (PROM1, PROM2, PROM3, and ClyRoM5) demonstrated diverse degrading capabilities, with *Pseudomonas protegens* (PROM2) standing out with a maximum efficiency of 91.5 ± 0.7% in hydrocarbon removal. Additionally, PROM2 showed notable removal of specific compounds such as Nonadecane (45.87 ± 5.87%). *Pseudomonas citri* PROM3 and *Acinetobacter guillouiae* ClyRoM5 strains also demonstrated high efficiency in the removal of various individual hydrocarbons, with *Pseudomonas protegens* PROM3 achieving 67.64 ± 1.43% in eicosane. These results highlight the potential of indigenous bacteria from uncontaminated soils for bioremediation applications, providing an effective and ecological alternative to hydrocarbon contamination in agricultural regions.

## Figures and Tables

**Table 1:** Biodegradation of diesel by different microbial isolates.

Microbial Isolation	Natural Source of Insulation	Diesel Biodegradation Yield	Reference
*Bacillus megaterium* MJ4	Seawater from Jiaozhou Bay, China.	Degradation rate at 26.54% in 5 days, using 10 g/L of diesel.	[7]
*Vibrio alginolyticus* MFI5	Diesel-contaminated seawater in Tanjung Jati, Madura Island, Indonesia.	Diesel degradation capacity up to 26.78% during 14 days of incubation at 10% initial diesel (*v/v*).	[8]
*Janthinobacterium lividum* AQ5-29 and *Pseudomonas fildesensis* AQ5-41	Antarctic soil of Signy Island.	Strains AQ5-29 and AQ5-41 removed 2.9 and 4.2 mg/mL of diesel, respectively, with biodegradation of C10 to C30 hydrocarbons ranging from 40 to 100% at 10 °C in less than 8 days.	[9]
*Acinetobacter calcoaceticus* and *Pseudomonas aeruginosa*	Wastewater samples with effluents from mechanical workshops, Burkina Faso (Africa).	Highest biodegradation rate at 65.53%, using both S2+S7 in diesel.	[10]

**Table 2:** Molecular characterization of the 16S rRNA gene of isolated microbial cultures.

Isolated Microbial Culture	Name	Similarity Strain	Pairwise Similarity (%)	Access Number	Link (accessed on 11 June 2024)
PROM1	*Priestia flexa*	NBRC 15715	99.59	PP886133	https://www.ncbi.nlm.nih.gov/nuccore/PP886133
PROM2	*Pseudomonas protegens*	CHA0	100	PP886146	https://www.ncbi.nlm.nih.gov/nuccore/PP886146
PROM3	*Pseudomonas citri*	OPS13-3	99.18	PP886148	https://www.ncbi.nlm.nih.gov/nuccore/PP886148
ClyRoM5	*Acinetobacter guillouiae*	CIP 63.46	100	PP892527	https://www.ncbi.nlm.nih.gov/nuccore/PP892527

**Table 3:** Tukey test between treatments of different isolated microbial cultures on diesel hydrocarbon removal.

Isolated Culture	MeanDiff	q Value	Prob
PROM1 and PROM3	−41.51	64.58	0.0 (*p* < 0.05)
PROM2 and PROM3	23.87	37.13	0.0 (*p* < 0.05)
PROM2 and PROM1	65.38	101.71	0.0 (*p* < 0.05)
ClyRoM5 and PROM3	−10.20	15.88	4.20 × 10^−1^
ClyRoM5 and PROM1	31.30	48.70	0 (*p* < 0.05)
ClyRoM5 and PROM2	−34.07	53.01	0 (*p* < 0.05)

## Data Availability

Data are contained within the article.
